# Biochar’s Leacheates Affect the Abscisic Acid Pathway in Rice Seedlings Under Low Temperature

**DOI:** 10.3389/fpls.2021.646910

**Published:** 2021-03-04

**Authors:** Jun Yuan, Jun Meng, Xiao Liang, E Yang, Xu Yang, Wen-fu Chen

**Affiliations:** ^1^Liaoning Biochar Engineering and Technology Research Center, Shenyang Agricultural University, Shenyang, China; ^2^Eastern Liaoning University, Dandong, China

**Keywords:** abscisic acid, biochar, cold stress, molecular docking, rice seedlings

## Abstract

Organic molecules of biochar’s leacheates are known to increase the cold resistance of rice seedlings. Yet, it remains unclear whether the organic molecules of biochar leacheates can interact with the abscisic acid (ABA) signaling pathway associated with low temperature. This study used experiments and bioinformatics (molecular docking) to determine which of the organic molecules of biochar’s leacheates could influence the ABA signaling pathway. Specifically, we investigated whether these molecules affected ABA, a plant hormone linked to cold resistance. The contents of endogenous ABA and its precursor carotenoids were determined under low-temperature stress (10°C) and treatment with different concentrations of biochar leacheates. With increased leacheate concentrations, the endogenous ABA and carotenoid contents also increased, as did the expression of ABA- and cold-related genes. When rice seedlings were instead treated with exogenous ABA, it also affected the above-measured indexes; hence, we surmised that certain water-soluble organic molecules of biochar could exert a similar effect as ABA. We first used gas chromatography/mass spectrometry (GC/MS) to identify the organic molecules in the biochar extract, and then we used molecular docking software Autodock to show how they interact. We found that the molecule (1R, 2R, 4S)-2-(6-chloropyridin-3-yl)-7-azabicyclo(2.2.1)heptane was simplified, as Cyah could dock with the ABA receptor protein OsPYL2 in rice, which shows Cyah in biochar is probably an analog of ABA, with a similar function. Based on these results, we conclude that organic molecules of biochar’s leacheates could enter into rice plants and interact with ABA-related proteins to affect the ABA signaling pathway, thereby improving the cold stress resistance of plants.

## Introduction

Biochar is the product of heating biomass in the absence of or with limited air to above 250°C in a process called charring or pyrolysis (Lehmann and Joseph, 2015). Biochar is often used as an additive to improve the quantity of the environment and amend the soil (Lehmann and Joseph, 2015), and such additions can reportedly enhance plant growth characteristics (Waqas et al., 2018). For example, biochar treatments increased both plant height and leaf size in tomato (Graber et al., 2010); treatment with attapulgite clay/yak dung (50/50) biochar resulted in the highest pasture yield and promoted the nutritional quality of grass (Rafiq et al., 2017); and biochar alone or in a co-application stimulated growth in halophyte plants, including their germination, root development, and biomass (Zheng et al., 2018).

Biochar can affect plant growth *via* several plausible mechanisms: (1) by improving soil and regulating the soil microbial environment, which indirectly or directly affects plant root growth and thus affects the whole plant (Lehmann and Joseph, 2015; Zheng et al., 2018); (2) by providing nutrients for plants to uptake (Wang et al., 2018); (3) by organic molecules on the surface of biochar that can promote or inhibit plant growth (Graber et al., 2010; Lievens et al., 2014; Gale et al., 2016; Yuan et al., 2017); and (4) by affecting endogenous plant hormones, which can impact plant development and physiology (Yang et al., 2015; French and Iyerpascuzzi, 2018; Waqas et al., 2018). Recently reported effects of biochar on plant hormones include changes to jasmonic acid levels in two rice varieties that altered their resistance to herbivory (Waqas et al., 2018) and evidence suggesting that biochar promotes growth, in part, *via* stimulation of the Gibberellic acid (GA) pathway (French and Iyerpascuzzi, 2018). The previous paper dealt with low temperature (cold), yet the relationship between biochar and abscisic acid (ABA)—which is closely related to low temperature—has not been reported on.

Low temperature may negatively impact agricultural crop productivity (Li et al., 2015). ABA is an essential phytohormone that not only regulates seed dormancy, germination, and seedling growth but also is involved in plant responses to environmental stresses, such as drought, high salinity, and chilling (He et al., 2014). In the presence of ABA, the ABA receptor pyrabactin resistance 1 (PYR1)/PYR1-like (PYL)/regulatory components of ABA receptor (RCAR) undergoes conformational changes and mediates interactions with the negative regulator type 2C protein phosphatase (PP2C), thus inhibiting their phosphatase activity, which then activates the positive regulator Class III SNF1-related protein kinase 2 (SnRK2s) to turn on downstream gene expression (Hubbard et al., 2010; Cao et al., 2013). The rice (*Oryza sativa*) ortholog of the ABA receptor in OsPYL/RCAR5 was recently identified as a positive regulator in seed germination and early seedling growth (Kim et al., 2012, 2014). He et al. (2014) determined the crystal structure of the ABA–OsPYL2–OsPP2C06 ternary complex, and the first structure of the ABA receptor in rice revealed a molecular mechanism of ABA sensitivity and phosphatase inhibition of OsPYLs (He et al., 2014).

Naturally occurring small molecules have long been foci for study due to their diverse biological activities (Bhuiya et al., 2017). One way to investigate these molecular and protein interactions is through molecular docking, which is now the most frequently used computational method for studying the interactions between organic molecules and biological macromolecules (Yang et al., 2015). In this context, docking is able to predict the preferred position of a ligand inside a receptor binding site (Ramirez and Caballero, 2018).

In previous work, we showed that biochar additions have a positive impact on cold stress resistance in rice plants (Yuan et al., 2017). Yet, it remains unclear whether the organic molecules of biochar can interact with the ABA signaling pathway associated with cold resistance. This study treated rice seedlings with different leacheates of biochar and combined the use of experiments and bioinformatics (molecular docking) to determine which of the organic molecules of biochar’s leacheates could influence the ABA signaling pathway. A mechanism was postulated: organic molecules of biochar’s leacheates can successfully connect with the ABA receptor protein, thereby affecting the ABA pathway of rice, which eventually fosters their resistance to cold.

## Materials and Methods

### Preparation of Biochar Leacheates and Exogenous Abscisic Acid Treatment

The biochar used in our experiments was generated from fast pyrolysis of rice husks (Shen-nong 9816), conducted at the Rice Research Institute of Shenyang Agriculture University, China. Rice husks were heated to 400°C at a rate of 10°C/min before the temperature was held constant for 1 h. The selected concentrations of biochar leacheates were 0, 1, 3, 5, and 10%. In the control group (0%, no biochar) we used 25 g of dry soil to cultivate the rice seedlings. To prepare the 1, 3, 5, and 10% concentrations of biochar leacheates, we, respectively, weighted 0.25, 0.75, 1.5, and 2.5 g of biochar and put them into separate beakers containing 50 ml of distilled water; these were stirred at 25°C for 72 h, and then filtered through a 0.22-μm sieve. All bacteria were removed from leacheates and soils by autoclaving at 121°C for 60 min, and all samples were stored at 4°C prior to further analysis.

For the exogenous ABA treatment, 0, 10, 20, and 30 mg of ABA was placed into respective Eppendorf (EP) tubes, with a little anhydrous ethanol (500 μl) added in to help dissolve them. Then, each mixture was transferred to a beaker containing 1,000 ml of distilled water and stirred well.

### Planting and Treatment of Rice Seedlings

The Japonica Super Rice “Shen-nong 9816,” a cultivar with strong resistance to stress and wide adaptability was used in this study, sourced from the Rice Research Institute, Shenyang Agricultural University, China. Its seeds were germinated in a culture dish with distilled water. Germinated seedlings were sown into small 7-cm-diameter pots containing 25 g of dry soil, and each pot received 50 ml of one of the five biochar leacheate concentrations. The properties of the soil are described in a previous paper (Yuan et al., 2017). All these samples were kept in growth chambers for 5 days at 28°C day and night but subject to a 12-h/12-h light/dark cycle at 75% relative humidity and the light intensity was maintained at 12,000–14,000 lux. The 5-day-old seedlings were kept at 10°C all day and night in another growth chamber (under the same light–dark cycle and relative humidity conditions) for 21 days to simulate the cold stress treatment. The four concentrations of the exogenous ABA treatment were sprayed onto the 5-day-old rice seedlings under a normal temperature (28°C) after which they were kept at 10°C for 21 days. Treatment time was based on when the plants developed obvious phenotypes at low temperatures or under control conditions. The experimental temperature and protocol used in this study follow those used by Challam et al. (2015). The response variables measured included plant height, dry weight, and root length for both the control and the four treatment groups. These data were used to evaluate the effects of low temperature on rice plant growth. Finally, some samples were kept in growth chambers for 7 days at 28°C day and night to serve as the normal temperature control.

### Measurement of Carotenoids and Abscisic Acid

Carotenoids were extracted from 0.1 g of rice seedling leaves *via* incubation for 72 h in 3 ml of 100% dimethyl sulfoxide at 65°C. The concentrations were calculated using an absorbance measurement of the extract at 480, 649, and 665 nm and the equations described in Pompelli et al. (2013). Endogenous ABA analysis was carried out using high-performance liquid chromatography (HPLC) (Agilent 1200 Series, United States) with an Agilent C18 column. The mobile phase was methanol:acetonitrile:acetic acid (60:5:35), the flow rate was 0.8 ml/min, and the injection volume was 10 microns, with samples detected at an absorbance of 262 nm (Cheng et al., 2013).

### Quantitative Real-Time PCR

Abscisic acid - and cold-regulated genes were identified in this study by using the National Center for Biotechnology Information (NCBI) database^1^, while the primers for the quantitative real-time PCR (qRT-PCR) genes were designed and amplified using the Primer 3 software. The qRT-PCR was carried out in a 20-μl reaction vessel that contained 10 μl of 2 × *TransScript*^®^ Top Green qPCR SuperMix (TransGen Biotech, China), 0.4 μl of passive reference dye, 0.4 μl of both forward and reverse primers, 4.2 μl of nuclease-free water, and 5 μl of diluted cDNA (1:10). The PCR amplification was performed using System LightCycler 480 equipment (Roche Applied Science, Germany), and the qRT-PCR procedure steps were 94°C for 30 s, followed by 45 cycles of 94°C for 5 s, 55°C for 15 s, and 72°C for 10 s. Values for gene expression were calculated following the method outlined by Ramakers et al. (2003) and used delta-delta Ct (Ramakers et al., 2003). The gene primers used for qRT-PCR are listed in Supplementary Table 1.

### Molecular Docking Analysis

We identified those proteins involved in ABA pathways as influenced by biochar organic molecules *via* comparison with the NCBI database and then utilized the Research Collaboratory for Structural Bioinformatics (RCSB) protein database to obtain their structures. The three-dimensional (3D) structures of small organic molecules were reported in our previous paper (Yuan et al., 2017), while specified target proteins and organic molecules for the docking analysis were determined using AutoDock tools v.1.5.6 in AutoDock software v.4.2 (Scripps Research Institute, United States) and the procedures recommended by Trott and Olson (2009).

### Statistical Analyses

Phenotypic parameters and ABA and carotenoid contents were derived from 30 biological replicates and were expressed as means ± SE. Expression of genes in rice plants were repeated independently for at least three times, and data are shown as means ± SE. All numerical data were analyzed using SPSS software (v17.0) and Microsoft Excel 2003. The use of ^∗^ and ^∗∗^ denotes different mean concentrations of biochar and ABA that exhibited significant differences at the *P* < 0.05 and *P* < 0.01 alpha levels when compared to the control group only.

## Results

### Biochar Affects the Abscisic Acid Signaling Pathway of Rice Seedlings

**FIGURE 1:**
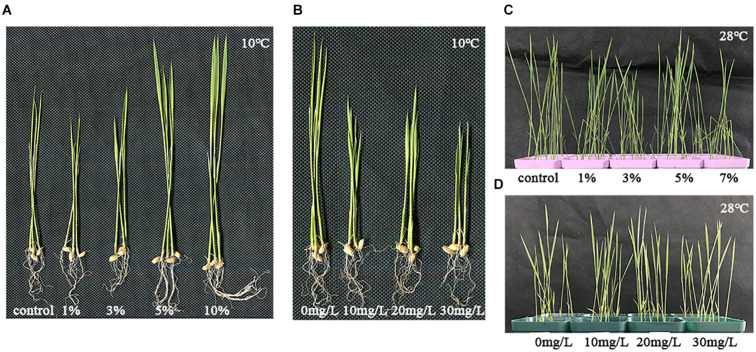
Phenotypes of plants treated with abscisic acid (ABA) and biochar leacheates under low and normal temperature. One part of each 5-day-old rice plants was then grown at 10°C for 21 days. This figure shows the different plant phenotypes that ensued under cold stress. **(A)** Rice plants were subjected to different concentrations of biochar leacheates (i.e., control, 1, 3, 5, and 10%). **(B)** Rice plants were subjected to different concentrations of ABA (0, 10, 20, and 30 mg/L). One part of rice plant was then grown at 28°C for 7 days, as the normal temperature control. **(C)** Biochar leacheates (i.e., control, 1, 3, 5, and 10%) and **(D)** rice plants were subjected to different concentrations of ABA (0, 10, 20, and 30 mg/L).

Several groups of experiments were conducted, and the results showed the same trend. A group of data and some rice seedlings were selected for the results and phenotypes presented in this paper. Rice plants treated with different concentrations of biochar leacheates (i.e., control, 1, 3, 5, and 10%) were grown in the same pots under well-watered conditions, and all of them developed the same phenotype after 5 days of growth at 28°C. While under cold stress, compared with the control; the 1% leacheate treatment reduced their plant height by 21.8% and root length by 14.58% (Table 1). However, greater leacheate concentrations (i.e., 3, 5, and 10%) led to continuous enhancement of rice plant growth (Figure 1A), though only the 5 and 10% concentrations significantly increased plant height (by 17.46 and 30.45%, respectively) and root length (by 22.05 and 34.91%, respectively). Changes in dry weight among leacheate concentrations were not significant, however (Table 1).

**TABLE 1:** Phenotypic parameters (mean ± SE) for one part of each 5-day-old rice seedling treated with biochar leacheates and grown at 10°C for 21 days.

Parameters	Biochar treatment concentrations				
	Control (0%)	1%	3%	5%	10%
Plant height (cm)	10.87 ± 0.60	8.50 ± 1.57*	10.93 ± 0.60	13.17 ± 2.02*	15.63 ± 0.42**
Root length (cm)	3.43 ± 0.25	2.93 ± 0.31	3.67 ± 0.25	4.4 ± 0.66**	5.27 ± 0.21**
Dry weight (mg)	0.0292 ± 0.0035	0.0235 ± 0.0037	0.0292 ± 0.0071	0.0309 ± 0.0085	0.0348 ± 0.0042

Asterisks indicate statistically significant differences from the control (n = 30, *P < 0.05, and **P < 0.01).

The phenotypic parameters of plants treated with different concentrations of exogenous ABA at 10°C are summarized in Table 2. Plant height in the 10, 20, and 30 mg/l ABA treatments was lower than in the 0 mg/l treatment; however, no significant changes were found in root length and dry weight relative to the control (Figure 1B and Table 2).

**TABLE 2:** Phenotypic parameters (mean ± SE) for one part (subsample of rice seedlings) of each 5-day-old rice seedling treated with ABA and grown at 10°C for 21 days.

Parameters	Exogenous ABA concentrations			
	0 mg/L	10 mg/L	20 mg/L	30 mg/L
Plant height (cm)	10.87 ± 0.60	6.83 ± 0.86**	7.47 ± 1.08**	6.05 ± 0.72**
Root length (cm)	2.47 ± 0.38	2.27 ± 0.25	2.1 ± 0.26	2.21 ± 0.28
Dry weight (mg)	0.0291 ± 0.0021	0.0255 ± 0.0061	0.0249 ± 0.0025	0.0231 ± 0.0019

Asterisks indicate statistically significant differences from the control (n = 30, *P < 0.05, and **P < 0.01). ABA, abscisic acid.

One part of rice seedlings was treated with biochar leacheates and grown at 28°C for 7 days as the normal temperature control. Compared with 0%, there were no significant changes found in plant height, root length, nor dry weight under the different concentrations of leacheates, except for the 3% leacheate treatment for plant height (Figure 1C and Table 3).

**TABLE 3:** Phenotypic parameters (mean ± SE) for one part of rice seedlings treated with biochar leacheates and grown at 28°C for 7 days.

Parameters	Biochar treatment concentrations				
	Control (0%)	1%	3%	5%	10%
Plant height (cm)	15.89 ± 0.15	15.78 ± 0.2635	14.30 ± 0.13**	16.28 ± 0.25	15.54 ± 0.23
Root length (cm)	5.34 ± 0.31	5.17 ± 0.48	5.25 ± 0.27	5.47 ± 0.20	5.50 ± 0.27
Dry weight (mg)	0.0322 ± 0.0014	0.0312 ± 0.0019	0.0338 ± 0.0020	0.0395 ± 0.0016	0.0332 ± 0.0022

Asterisks indicate statistically significant differences from the control (n = 30, *P < 0.05, and **P < 0.01).

However, there were no significant changes found in plant height, root length, and dry weight under different concentrations of exogenous ABA treatments (Figure 1D and Table 4).

**TABLE 4:** Phenotypic parameters (mean ± SE) for one part of rice seedlings treated with ABA and grown at 28°C for 7 days.

Parameters	Exogenous ABA concentrations			
	0 mg/L	10 mg/L	20 mg/L	30 mg/L
Plant height (cm)	12.61 ± 0.49	13.01 ± 0.93	13.32 ± 1.34	12.41 ± 0.75
Root length (cm)	2.48 ± 0.14	2.70 ± 0.15	2.77 ± 0.47	2.78 ± 0.21
Dry weight (mg)	0.0293 ± 0.0044	0.0329 ± 0.0031	0.0367 ± 0.0035	0.0286 ± 0.0028

Asterisks indicate statistically significant differences from the control (n = 30, *P < 0.05, and **P < 0.01). ABA, abscisic acid.

### Quantitative Real-Time PCR Analysis

**FIGURE 2:**
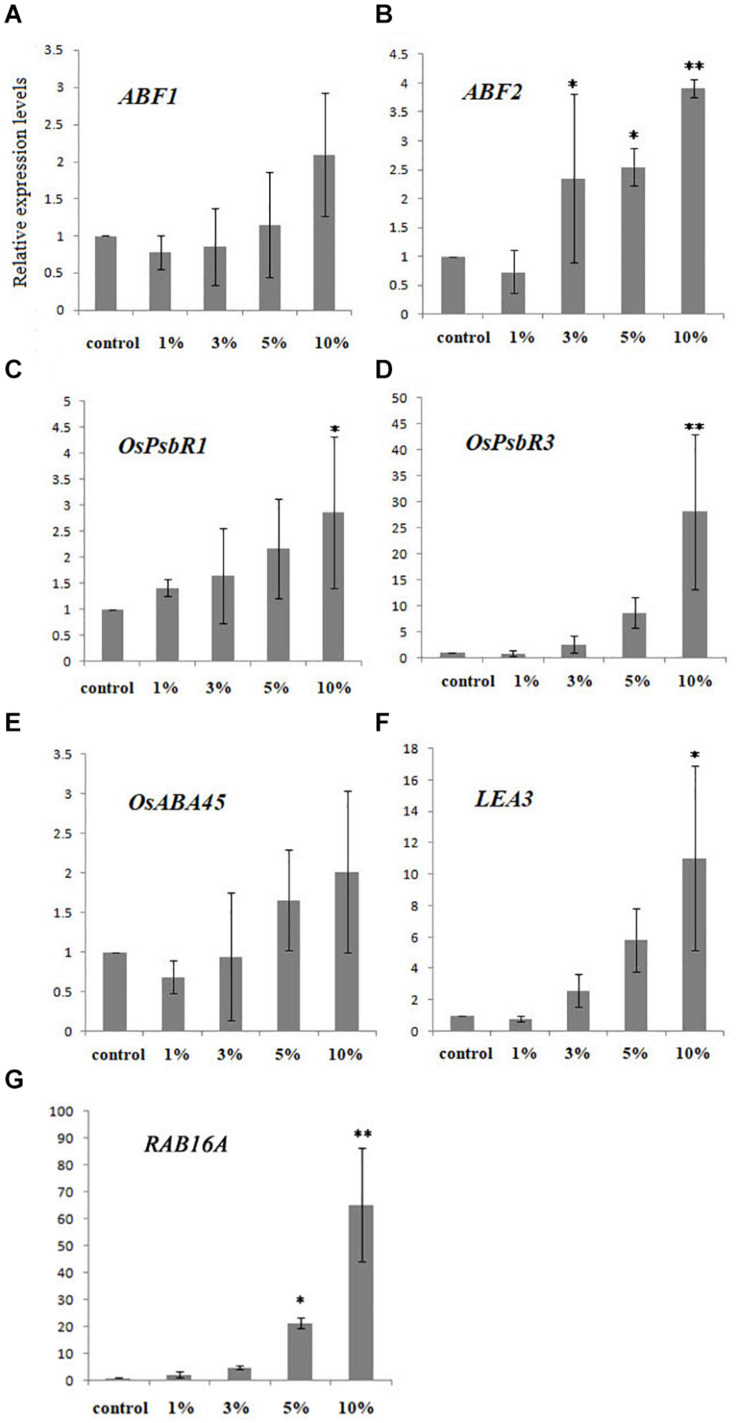
Expression of ABA -and cold-related genes in rice plants treated with five concentrations (%) of biochar leacheate. **(A)** *ABF1*. **(B)** *ABF2*. **(C)** *OsPsbR1*. **(D)** *OsPsbR3*. **(E)** *OsABA45*. **(F)** *LEA3*. **(G)** *RAB16A*. Bars are mean ± SE, while asterisks indicate statistically significant differences from the control (*n* = 3, ^∗^*P* < 0.05, ^∗∗^*P* < 0.01).

To further elucidate the influence of biochar on cold tolerance, we selected seven important ABA- and cold-related genes (i.e., *ABF1*, *ABF2*, *OsPsbR1*, *OsPsbR3*, *OsABA45*, *LEA3*, and *RAB16A*) known for their involvement in the ABA and cold signaling pathways. Relative expression analysis using qRT-PCR revealed that the proportion of these transcription factors changed depending on the biochar leacheate concentrations applied (i.e., 1, 3, 5, and 10%) when compared with the control. In this experiment, relative to the control, the expression of *OsABF1* under high concentrations of biochar leacheates (5 and 10% treatments) was upregulated, whereas it was downregulated at low concentrations (1 and 3% treatments); however, no significant differences were detected (Figure 2A). Likewise, *OsABF2* expression levels in the treatments with 3, 5, and 10% biochar leacheates were upregulated but downregulated in the 1% concentration treatment (Figure 2B). The expression of *OsPsbR1* in all biochar leacheate treatments except that of 10% was similar to that of the control (Figure 2C). *OsPsbR3* expression was significantly upregulated under greater biochar leacheate concentrations (Figure 2D), while *OsABA45* was upregulated only under the 5 and 10% concentrations of biochar leacheates and downregulated in the other two treatments (Figure 2E). The expression of *LEA3* was significantly upregulated and downregulated in the 10 and 1% concentration treatments, respectively (Figure 2F). With more biochar applied, the expression level of *RAB16A* gradually increased, differing significantly from that of the control in the 5 and 10% concentration treatments (Figure 2G).

**FIGURE 3:**
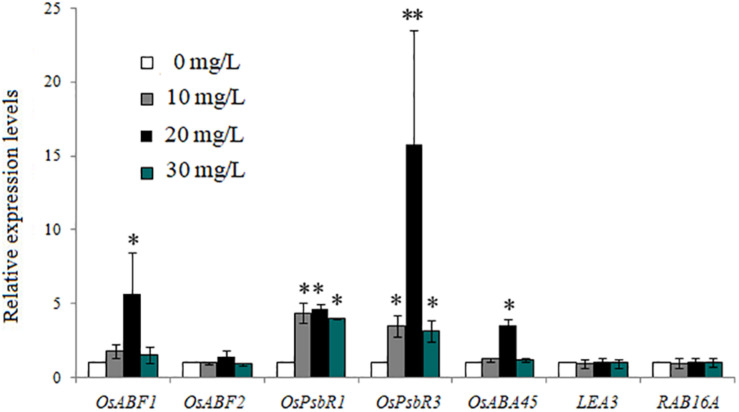
Expression of abscisic acid (ABA)- and cold-related genes in rice plants treated with the three concentrations of exogenous ABA. Bars are mean ± SE, while asterisks indicate statistically significant differences from the control (*n* = 3, ^∗^*P* < 0.05, ^∗∗^*P* < 0.01).

Figure 3 shows the expression levels of the same seven genes in rice seedlings treated with different concentrations of exogenous ABA. Compared with the control (treated with 0 mg/L exogenous ABA), in rice seedlings treated with 10, 20, and 30 mg/L exogenous ABA, the expression levels of *OsABF1*, *OsABF2*, *OsABA45*, *OsPsbR1*, and *OsPsbR3* were gradually upregulated (except the expression levels of *OsABF2* in 30 mg/L exogenous ABA), while the expression of *LEA3* and *RAB16A* genes was not induced by 10 nor 30 mg/L, but slightly upregulated by 20 mg/L ABA.

### Exogenous Abscisic Acid Treatment Analysis

**FIGURE 4:**
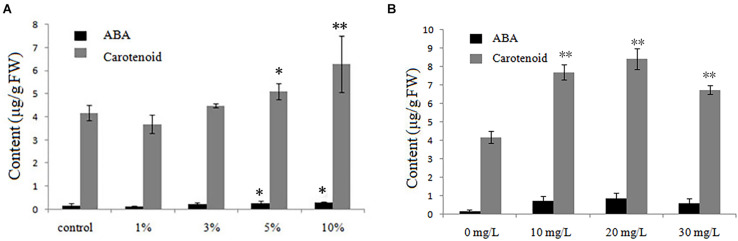
ABA and carotenoid contents of rice plants treated with, **(A)** five concentrations (%) of biochar leacheate and **(B)** four concentrations of exogenous ABA. Bars are mean ± SE, while asterisks indicate statistically significant differences from the control (*n* = 30, ^∗^*P* < 0.05, ^∗∗^*P* < 0.01).

Under low-temperature stress, 21-day-old rice seedlings grown with different biochar leacheate concentrations (control, 1, 3, 5, and 10%) or different exogenous ABA concentrations (0, 10, 20, and 30 mg/L) were sent to the Wanze biotechnology company (Shenyang, China) to measure their contents of ABA and ABA-precursor substances (carotenoid). Compared with the control, both ABA and carotenoid were lower in rice seedlings treated with 1% biochar leacheate, but with greater leacheate concentrations (3–10%), they gradually increased (Figure 4A). The ABA and carotenoid contents were promoted by ABA applied to the plants, reaching their maximum value at 20 mg/L (Figure 4B).

### Docking Analysis

In our prior study, we had used the gas chromatography/mass spectrometry (GC/MS) method to extract 20 organic molecules from the surface of biochar, of which 14 kinds of organic molecules with a relatively small relative molecular weight were used for subsequent molecular docking (Yuan et al., 2017). Here, we used the Plant Metabolic Pathway (PMN) database to identify the potential biological activities of these candidate compounds (Table 5).

**TABLE 5:** Candidate organic molecules obtained from biochar surface extracts.

Name	Potential biological activity
6-(Methylthio)hexa-1,5-dien-3-ol	No function has been reported
Formamide, N, N-diethyl-	No function has been reported
1-Oxa-4-azaspiro(4.5)decan-4-oxyl, 3,3-Dimethyl-8-oxo-	No function has been reported
2-Propanamine, N,N-dimethyl-	Involved with enzyme compensation system reaction
Ethanamine, N-pentylidene-	Takes part in some chemical reactions
Acetamide, N,N-diethyl-	Involved with enzyme compensation system reaction
Cyclopentanone, 2-(1-methylpropyl)-	No function has been reported
Cyclopentane, 1,2,3-trimethyl-	Biosynthesis of jasmonic acid
Pyrrole, 2-(4-methyl-5-cis-phenyl-1,3-oxazolidin-2-yl)-	Four pyrrole synthesis pathways Four pyrrole degradation pathways
1,2-Dimethylaziridine	No function has been reported
(1R,2R,4S)-2-(6-chloropyridin-3-yl)-7-azabicyclo(2.2.1)heptane (Cyah)	Takes part in most chemical reactions
2-Acetyl-5-methylfuran	No function has been reported
Pyridine	Participates in some conventional chemical reactions
*Trans-*2,4-Dimethylthiane, S,S-dioxide	Involved with enzyme compensation system reaction

**FIGURE 5:**
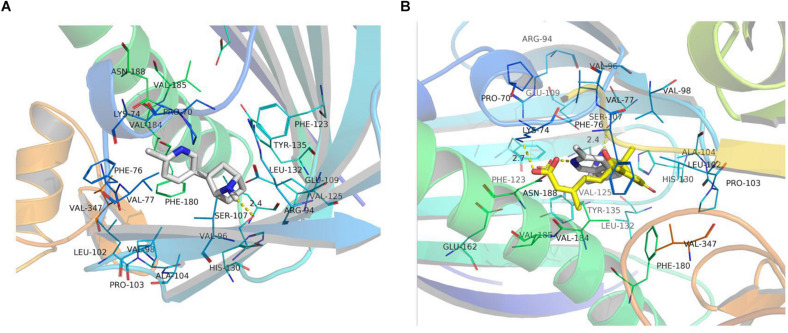
Docked OsPYL2 active site with Cyah and abscisic acid (ABA). **(A)** Cyah docked with the OsPYL2 active site. **(B)** Cyah and ABA docked with OsPYL2. These images were drawn using the software program PyMOL.

We searched for the ABA-related receptor protein in the RCSB database and found that the protein OsPYL2 (ID:4OIC) from rice had a known 3D structure (He et al., 2014). All the candidate organic molecules (Table 5) were docked with the OsPYL2 protein, and the organic molecule (1R, 2R, 4S)-2-(6-chloropyridin-3-yl)-7-azabicyclo[2.2.1]heptane was simplified as Cyah (white molecules in Figure 5A), which could be successfully docked with protein OsPYL2. Cyah is linked to the amino acid residue SER-107 of the protein by a hydrogen bond (yellow dashed line). The binding mode of the OsPYL2 protein to the original ligand ABA (yellow molecule, depicted in Figure 5B) is also a hydrogen bond, while ABA is linked to the amino acid residue LYS-74 in the protein by hydrogen bond. Because the association of Cyah and OsPYL2 is *via* hydrogen bonding, like that of ABA and OsPYL2, we reasonably speculate that the function of Cyah may be similar to that of ABA.

## Discussion

High concentration of biochar enhanced the growth of a bean under saline condition, which may have contributed to the reduction of Na uptake and enhancement of K, Ca, and Mg contents (Farhanqi-Abriz and Torabian, 2018), and application is known to preserve rice pollen given high-temperature stress (Fahad et al., 2015). Our results also reveal high concentrations of biochar leacheates led to enhancement of rice plant growth under low-temperature stress (Figure 1A). The heights of the plants treated with 10, 20, and 30 mg/L exogenous ABA were less than those lacking ABA and those of 20 mg/L treatment were slightly better (Figure 1B). This phenotype (Figure 1B) may arise because although ABA apart from a gradual increase in a certain low concentration can affect cold resistance in plants, its primary role is to slow plant growth (Mega et al., 2015). In this way, when temperatures are low and the plant grows slowly, it can better protect itself from this temporary stress (Mega et al., 2015). Under low temperature, both biochar and ABA can influence the growth and development of rice. However, within a certain concentration range, the biochar does not have the inhibiting effect of ABA (Figures 1A,B). Biochar and ABA can play their roles better under adverse environmental conditions (Kim et al., 2014; Fahad et al., 2015). Thus, in our study, there were no significant changes found in plant phenotypes under different concentrations of biochar leacheates and exogenous ABA treatments in the normal temperature control (Figures 1C,D).

A number of basic leucine zipper (bZIP) transcription factors are known to function in stress signaling in plants, but to date, few have been functionally characterized in rice: the *OsABF1* and *OsABF2* genes do encode a bZIP transcription factor (Hossain et al., 2010a, b). Their expression in seedling shoots and roots is reportedly induced by various abiotic stress treatments, such as anoxia, salinity, drought, oxidative stress, cold, and ABA (Hossain et al., 2010a, b). However, under the same low temperature, the expression of *OsABF1* and *OsABF2* genes within a plant may differ, since they can each respond to ABA (Figures 2A,B).

PsbR is known as the 10-kDa Photosystem II polypeptide. Although this plant PsbR is thought to play important roles in photosynthesis, little is actually known about its contribution to abiotic stress resistance (Li et al., 2017). In a recent study, the *OsPsbR1* gene was upregulated in response to cold stress, while the upregulation of *OsPsbR3* gene was observed when plants were treated with ABA (Li et al., 2017). We found that the expression of *OsPsbR1* in each biochar treatment was similar to that of the control, except under the 10% concentration treatment (Figure 2C), while that of *OsPsbR3* increased with all leacheate concentrations tested (Figure 2D). When ABA changes, the expression level of *OsPsbR3* gene also changes, indicating that the surface substances of biochar may affect the endogenous ABA in rice. The *OsABA45* gene in rice seedlings can be induced by low temperature, dehydration, high salt, and ABA (Rabbani et al., 2003); *LEA3* and *RAB16A* genes are associated with stress and can be induced by ABA (Zou et al., 2008; Hossain et al., 2010b). At the same low temperature, the expression of *OsABA45*, *LEA3*, and *RAB16A* genes differed in response to ABA (Figures 2E–G). Therefore, we speculate that substances in the biochar leacheates can affect the ABA pathway.

As a phytohormone, ABA is extensively involved in plant responses to abiotic stresses, such as drought, low temperature, and osmotic stress. In response to cold stress, plants usually accumulate an increased amount of ABA, and many stress-inducible genes are regulated by the endogenous ABA that accumulates during conditions of stress (Shinozaki et al., 2003). We found that a high concentration of biochar leacheates could improve the content of ABA and its precursors carotenoids in rice seedlings (Figure 4A), as well as the expression of ABA and the cold-related genes *OsABF1*, *OsABF2*, *OsABA45*, *OsLEA3*, *RAB16A*, *OsPsbR1*, and *OsPsbR3* (Figure 2). This result indicates that adding more biochar leacheate can induce endogenous ABA biosynthesis in rice seedlings, and this increased ABA could influence corresponding biological functions to help plants resist cold stress. High concentrations of biochar could affect the ABA signaling pathway, and exogenous ABA could also affect the ABA signaling pathway, indicating that some ABA analogs may exist on the surface of biochar. These ABA analogs likely caused a series of physiological and biochemical processes related to ABA and cold resistance within a certain concentration range, which eventually promoted the cold resistance of rice plants.

Working with tomato, Graber et al. (2010) found that biochar treatments positively enhanced its plant height and leaf size without any effect on its flower and fruit yield (Graber et al., 2010). Yet these positive impacts of biochar on plant responses were not due to direct or indirect effects on plant nutrition *per se*, as there were no differences between control and treatments in their leaf nutrient contents (Graber et al., 2010). Therefore, those authors considered that the organic molecules in biochar were impacting the growth of crops. In the absence of interference from other factors, we also think that the organic molecules of biochar’s leacheates are crucial for altering the growth of rice seedlings under low-temperature stress. To explore the mechanism by which such organic molecules could influence rice seedlings cold tolerance, we recently identified them (Yuan et al., 2017). In researching the direct effects of biochar on plants, most studies have now identified organic molecules from biochar *via* GC/MS and then determined whether they can affect plants’ growth and by which possible mechanism (Graber et al., 2010; Gale et al., 2016).

We used biochar leacheates to eliminate other interferences, so we believe that the organic molecules contained in biochar are indeed an important factor influencing the growth of rice seedlings under low temperature. But admittedly, the underlying mechanism of these organic molecules is not yet known. In recent years, the interaction between molecules and proteins has become a hot research topic. In plants, the function of organic molecules can include hormone response, signal transduction, or ligand interaction with proteins to exercise a series of related biological functions (Antunes et al., 2011; Yang et al., 2015; Bhuiya et al., 2017; Haruta and Sussman, 2017). Therefore, we put forward an experimental hypothesis: organic molecules of biochar’s leacheates can enter cells of the plant and interact with corresponding proteins in them, thereby driving a series of physiological and biochemical reactions that enable plants to resist the cold. We suspected our identified organic molecules may have interacted mechanistically with stress or ABA-related proteins to generate the cold stress effects we observed.

The direct homologous receptor OsPYL/RCAR5 has been shown to positively impact the growth of rice seedlings (Kim et al., 2012, 2014). He et al. (2014) identified the structure of OsPYL2 in rice, and its 3D structure was found in the RCSB database (He et al., 2014). We concluded that the organic molecule Cyah in biochar could be successfully docked with OsPYL2 (Figure 5). The organic molecule Cyah has been recorded in the PMN database given its participation in a variety of chemical reactions (Table 5), but whether it interacts with the ABA receptor protein remains is unknown. Nevertheless, the interaction between the ABA ligand and ABA receptor protein can reduce abiotic stress (Cao et al., 2013). The ABA analogs and ABA ligands are the same as ABA receptor proteins, and the interaction between ABA analogs and ABA receptor proteins can prompt plant responses to abiotic stress, thus indicating that ABA analogs also function much like ABA ligands. Cyah and ABA are the same as the ABA receptor protein OsPYL2, in that they are combined with hydrogen bonds, so Cyah may also be analogs to ABA, with the same function as ABA in regulating OsPYL2 to produce a series of related effects. A plausible mechanism is that the organic molecule Cyah in biochar combines with OsPYL2 of rice, and the combination of PP2C and Cyah-OsPYL2 then inhibits the activity of PP2C itself; this activates SnRK2 and regulates the ion channel, second messenger, and the expression of related ABA genes, which together improves the cold resistance of plants.

## Data Availability Statement

The original contributions presented in the study are included in the article/Supplementary Material, further inquiries can be directed to the corresponding author/s.

## Author Contributions

JY designed and carried out the experiments, analyzed the results, and wrote the manuscript. JM, YE, XL, XY, and WC designed the experiments. All authors contributed to the article and approved the submitted version.

## Conflict of Interest

The authors declare that the research was conducted in the absence of any commercial or financial relationships that could be construed as a potential conflict of interest.

## Footnotes

1 www.ncbi.nlm.nih.gov
