# Supplementary material for: Biochar’s Leacheates Affect the Abscisic Acid Pathway in Rice Seedlings Under Low Temperature
Source: Front Plant Sci. 2021 Mar 4;12:646910. doi: 10.3389/fpls.2021.646910 (PMC7970111; doi:10.3389/fpls.2021.646910)
Supplement: Supplementary file 1 [file Table_1.DOCX]

Supplementary Material

**Supplementary Table 1** Primers used in this study

| Name | Sequence (5’– 3’) |
| --- | --- |
| *RAB16A-F*  *RAB16A-R*  *LEA3-F*  *LEA3-R*  *OsABA45-F*  *OsABA45-R*  *OsPsbR1-F*  *OsPsbR1-R*  *OsPsbR3-F*  *OsPsbR3-R*  *OsNAC-F*  *OsNAC-R*  *Actin-F*  *Actin-R*  *OsABF1-F*  *OsABF1-R*  *OsABF2-F*  *OsABF2-R* | CAACGCTCCGGCAGCTCCAG  ATGCTGCTGCTCGCCCTTGT  AGACCTCCAGCACGTCGCAG CCCCCAATTTCTGGAGAATC  AGAGAGGGGACAGCCCGAT  GAGGCTCAGCTTCCCCATCGC  GTTAAGAGATCATTTGCATT  TTACATTCCTAGTGAAGATT  GGCTTTGCATCCATCCATCCA  CCGGTGGGAGACCATTCCT  TGTACGGAGAGAAGGAGTGGT  TCATCCAACCTGAGGCTGTTC  GATATGGAGAAGATCTGGCA  TAGCTCTTCTCCACGGAGGA  TCGCACACGGCATCGGATCT  AGTTGCGTGACCAGCGACTC  AAGCTTATGGAGTTGCCGCGGATGGG  GAATTCTCAGCATGGACCAGTCAGTGT |
